# Novel Multicentric Hepatic Lymphoma with Extrahepatic Biliary Obstruction Associated with Duodenal Perforation in a Cat

**DOI:** 10.1155/2021/5808886

**Published:** 2021-12-09

**Authors:** Isabella Hildebrandt, Adam Rudinsky, Valerie Parker, Jenessa Winston, Alexandra Wood, Samantha Evans, James Howard

**Affiliations:** ^1^Department of Veterinary Clinical Sciences, The Ohio State University, 601 Vernon L Tharp St., 43210 Columbus, OH, USA; ^2^Comparative Hepatobiliary and Intestinal Research Program, The Ohio State University, Columbus, OH, USA

## Abstract

An 11-year-old male castrated domestic shorthair cat was presented for evaluation due to clinical deterioration and potential extrahepatic biliary obstruction (EHBO). Further investigations confirmed EHBO and revealed severe and previously unreported comorbidities. On initial examination, the cat was markedly icteric with a poor body condition score and severe muscle wasting. Serum chemistry and complete blood count showed evidence of cholestasis and anemia. Primary diagnostics and therapeutics targeted these abnormalities. Abdominal ultrasound revealed peritoneal effusion, multifocal mixed echogenic hepatic and splenic foci, small intestinal thickening, cholelithiasis, choledocholithiasis, and common bile duct and pancreatic duct dilation with evidence of obstruction. Peritoneal effusion cytology confirmed septic peritonitis. Hepatic and splenic cytology was consistent with lymphoma. Based on these results, euthanasia was elected by the owners of the animal. Necropsy confirmed the ultrasound diagnoses, septic peritoneal effusion associated with a duodenal perforation, multiorgan lymphoma, and common bile duct carcinoma. Flow cytometry classified the lymphoma as a double-negative phenotype of T-cell lymphoma (CD3+ and CD5+, but CD4- and CD8-) present in the duodenum and liver and suspected in the spleen which has previously not been reported in cats. This case report documents a cat with EHBO caused by multiple disease processes including a novel T-cell lymphoma phenotype, biliary carcinoma, duodenal perforation and septic abdomen, and choleliths, as well as inflammatory hepatobiliary disease.

## 1. Introduction

Extrahepatic biliary obstruction (EHBO) is a multifactorial syndrome involving pancreatic, gastrointestinal, and hepatobiliary organs systems. The obstruction of bile flow from the biliary system into the duodenum can be associated with various conditions. In cats, the most commonly documented causes are typically either neoplastic or inflammatory in origin. Specific etiologies include lymphoma, adenocarcinoma, mast cell tumors, pancreatitis, cholangiohepatitis, cholecystitis, inflammatory bowel disease, and cholelithiasis [1–6]. Less commonly reported causes include diaphragmatic hernia, foreign body obstruction, parasitic infection, and congenital gallbladder anomalies [1, 5–8]. The exact pathogenesis of EHBO in cats is not fully understood and likely is variable between cases.

The case described herein typifies the medical proclamation known as “Hickam's dictum” that states a patient can have many different contributing medical conditions. This patient exhibited a unique combination of comorbidities potentially contributing to the clinical EHBO presentation, including a novel phenotypic lymphoma, duodenal perforation and septic abdomen, biliary carcinoma, distal pancreatic ductal occlusion caused by concurrent choledocholithiasis, pancreatic calculi, severe hepatopathy, steroid administration, and neoplasia. The case presentation, diagnostic history, and necropsy findings will be reviewed. The comorbidities associated with EHBO and surgical interventions will also be discussed as it relates to future feline awareness, potential treatment strategies, and recognition of multiple severe and previously undocumented concurrent comorbidities.

## 2. Case Presentation

An 11-year-old male castrated domestic shorthair cat was presented through the emergency service at an academic hospital for continued lethargy, weight loss, and hyporexia. Historically, the patient was diagnosed with *Escherichia coli* cholangitis and a two-year duration of cholangiohepatitis and suspected inflammatory bowel disease. These were managed with a combination of antimicrobials, prednisolone, hepatoprotectants, and weekly cobalamin (vitamin B12) injections. Prednisone was discontinued two weeks prior to presentation with worsening clinical signs following the cessation of the steroid administration.

One month prior to referral, the patient had increasing lethargy and weight loss. Complete blood count and chemistry revealed a nonregenerative anemia (packed cell volume 17%, ref. 10.3-52.3; reticulocyte 8.5 × 10^9^/L, ref. 3-50), leukocytosis (55.56 × 10^9^/L, ref. 2.87-17.02), neutrophilia (50.99 × 10^9^/L, ref. 2.3-10.29), thrombocytopenia (84 × 10^9^/L, ref. 151-600), mildly elevated alkaline phosphatase (ALP) (170 IU/L, ref. 11-111), and increased total bilirubin (2.0 mg/dL, ref. 0.0-0.9). A complete blood count was repeated 1-2 weeks later that showed a static anemia (packed cell volume 21.4% and 22.6%, ref. 12.2-52.7), persistent leukocytosis (57.8 × 10^9^/L and 44.4 × 10^9^/L, ref. 3.9-19) with a left shift (neutrophils 52.02 × 10^9^/L and 38.76 × 10^9^/L, ref 2.62-15.17), persistent mild monocytosis (2.89 × 10^9^/L and 1.34 × 10^9^/L, ref. 0.04-0.53), resolved thrombocytopenia (165 K/*μ*L and 398 K/*μ*L ref. 155-641), and an eosinophilia (2.31 and 2.73 × 10^9^, ref. 0.17-1.57). An abdominal ultrasound performed prior to its emergency visit showed a partial common bile duct obstruction secondary to choledocholithiasis; the common bile duct was dilated at 1.2 cm. Medical management was elected but little improvement was noted.

### 2.1. Investigations

At the time of presentation to the authors' institution, the cat was severely icteric with a poor body condition score (2/9) and severe muscle wasting. A grade IV/VI left parasternal cardiac murmur was auscultated. The remainder of the physical exam was within normal limits. Venous blood gas and electrolytes were within normal limits. Complete blood count confirmed a nonregenerative anemia (24%, ref. 10, 12–29), leukocytosis characterized by a neutrophilia (leukocytes 44.4 × 10^9^/L, ref. 2.3-18.4, neutrophils 41.3 × 10^9^/L, ref. 30-61), and a mild thrombocytosis (528 × 10^9^/L, ref. 128-444). A serum chemistry profile showed further increase in liver enzymes (ALT 355 IU/L, ref. 31-115; AST 124 IU/L, ref. 10–29, 31–44; ALP 461 IU/L, ref. 30-61; GGT 3 IU/L, ref. 0-1). Total bilirubin was increased (4.23 mg/dL, ref. 0-0.1). Hyperproteinemia (8.6 g/dL, ref. 5.3-8.5) was characterized by hyperglobulinemia (6.1 g/dL, ref. 2.2-5.3) and hypoalbuminemia (2.5 g/dL, ref. 2.8-4.1). Urinalysis showed isosthenuria (USG 1.014), proteinuria (1+ mg/dL), and bilirubinuria (2+).

Three view thoracic radiographs showed a diffuse bronchial pattern, sternal lymphadenopathy, and cardiomegaly with no signs of heart failure. There was decreased peritoneal serosal detail in the cranial abdomen noted indicating effusion. An echocardiogram was performed that showed mild pericardial effusion and no other structural flow abnormalities. Abdominal ultrasound showed severe cholecystitis, cholehepatitis, and choledochitis. A hyperechoic hilar liver tumor and mixed echogenic splenic pathology were also found. There was biliary debris noted in the proximal common bile duct and two choledocholiths at the level of the duodenal papilla. The combined terminal portion of the cystic duct and proximal pancreatic duct was severely dilated measuring 1.1 cm with intramural emphysema. The kidneys showed nonspecific cortical nephropathy and chronic cortical infarction with left nephrolithiasis. A mild amount of peritoneal effusion was confirmed and sampled.

The liver and spleen were aspirated under ultrasound guidance, and prepared slides were stained with modified Wright-Giemsa for cytopathology review. In both organs, there was a moderate to marked expansion of intermediate to large lymphocytes (nucleus 1-1.5× the size of a neutrophil) with round to slightly indented nuclei, smooth chromatin, and inapparent nucleoli. These lymphocytes had a scant rim of pale cytoplasm without visible granules. Taken together, this finding was interpreted as probable lymphoma in both the spleen and liver, and flow cytometry was recommended for immunophenotyping. Cytology of the liver also revealed moderate suppurative inflammation and moderate hepatocellular vacuolation consistent with lipid. Cytology of the spleen also reveals mild plasma cell hyperplasia and mild extramedullary hematopoiesis.

**Figure 1:**
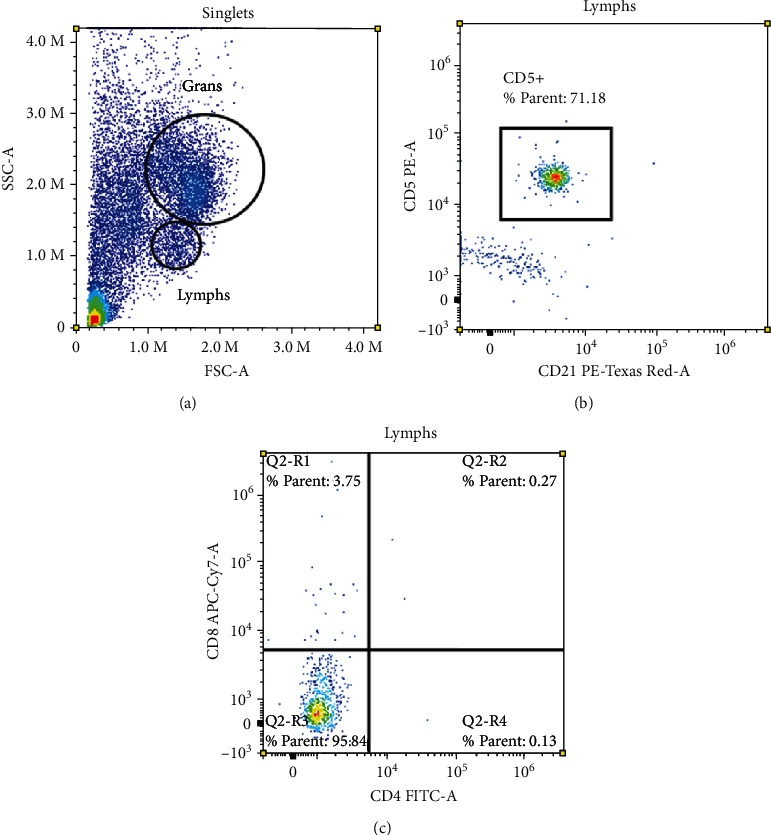
Flow cytograms of a liver aspirate sample. (a) Two scatter gates are drawn around granulocytes (larger, more complex cells) and lymphocytes (smaller, less complex cells). (b) Approximately 71% of the cells in the lymphocyte gate are positive for CD5 (T-cell marker) and negative for CD21 (B-cell marker). (c) The vast majority of these cells (~96%) are negative for the T-cell subset antigens CD4 and CD8.

A sample of hepatic tissue was aspirated into liquid media (90% normal saline, 10% feline serum) and processed for flow cytometry using routine methods, as previously described [29]. Cells were stained with a single panel of antibodies recognizing canine CD21 (clone CA2.1D6), canine CD18 (clone CA1.4E9), and feline CD5 (clone FE1.1B11), CD4 (clone vpg34), and CD8 (clone vpg9). Flow cytometry was performed on a Cytek Northern Lights flow cytometer, and data analysis was performed using SpectroFlo software (Cytek Biosciences, Bethesda, MD). Flow cytometry revealed a predominant population of granulocytes consistent with phenotypically normal neutrophils, confirming the finding of suppurative inflammation by cytology. In the lymphocyte scatter gate, there was a marked expansion of intermediate-sized lymphocytes expressing CD5+ (a T-cell marker) but no lineage-specific subset markers (CD4- and CD8-). This finding was considered diagnostic for lymphoma based on aberrant T-cell marker expression, in conjunction with the cytology findings (Figure 1).

### 2.2. Treatment

During hospitalization, the patient was maintained on hypotonic saline (0.45% NaCl) and started on enrofloxacin (5 mg/kg IV q24h). No other treatments were provided during the intake or diagnostic portion of the cat's provided care.

### 2.3. Outcome

Prognosis was considered grave with medical therapy or surgical treatment due to the severity of the hepatobiliary and pancreatic disease and concurrent diagnosis of multicentric lymphoma. Humane euthanasia was elected given the comorbidities, patient discomfort, and clinical decline. A full necropsy with histopathology was performed, and findings supported a severe multifactorial disease pathogenesis.

**Figure 2:**
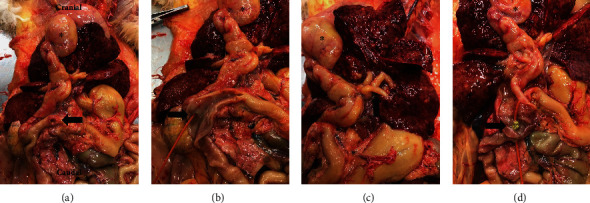
Necropsy images illustrating diffuse icterus, mottled liver, and severely dilated biliary system. Asterisk (^∗^) denotes the gallbladder in all images. (a) The arrow shows the full thickness duodenal ulceration. (b) The duodenum was opened, and a red rubber was passed through the major duodenal papilla (arrow) into the severely dilated common bile duct. (c) The arrow denotes the severely dilated hepatic ducts joining the cystic duct into the common bile duct. (d) The common bile duct was incised showing purulent discharge and a large stone lodged at the major duodenal papilla (arrow).

The gross pathology examination showed marked dilation of the gallbladder and common bile duct (Figure 2). There was diffuse viscous purulent material within the lumen of the gallbladder and common bile duct. Culture of the bile and biliary tissue showed growth of *E. coli*, *enterococcus*, *faecium*, and *capnocytophaga* spp. The pancreatic ducts were markedly dilated. Choleliths were noted in the cystic and common bile duct indicating multifocal cholelithiasis and choledocholithiasis. There was a large stone at the level of the major duodenal papilla. Pancreatic calculi were also noted. Stone analysis for both the biliary and pancreatic ducts could not be performed due to the laboratory restrictions during the COVID-19 pandemic. However, inhouse analysis suspected calcium bilirubinate for the choleliths; no analysis was performed on the pancreatic stones. The common bile duct was minimally patent due to wall thickening. A focal full-thickness duodenal perforation measuring 4 mm in diameter was found at the level of the duodenal papilla. The liver was diffusely infiltrated with multifocal to coalescing white tissues and had a focal mass that was molted tan to red and friable on cut section. The spleen had no gross abnormalities. A solitary tan pulmonary nodule was also noted.

Following necropsy, histopathology of the liver revealed a neoplastic round cell (lymphocyte) population effacing portal triads and expanding hepatic sinusoids with marked multifocal ductular reaction and periportal edema. There was also marked multifocal neutrophilic cholangitis with circumferential fibrosis and edema around bile ducts. The common bile duct wall showed cholangiocellular carcinoma arising in severe widespread cholangiocellular hyperplasia on histopathology with positive CK7 staining (carcinoma marker). Neoplastic cells surrounding the portal triads were immunopositive for CD3 by immunohistochemistry (IHC), confirming the finding of T-cell lymphoma by flow cytometry. The same neoplastic cell population was observed throughout the duodenal mucosa and extending into the underlying lamina propria. It was difficult to determine whether lymphoma was also present in the spleen by histopathology, as the CD3-positive cells did not form discrete masses and there may be large amounts of circulating T-cells normally in the red pulp. Unfortunately, feline CD4 or CD8 immunostaining is not available by IHC as these antibodies do not bind to formalin-fixed tissue; so, the double-negative phenotype could not be confirmed in the spleen. Lymphoma was not observed by histopathology in any regional lymph nodes. Histopathology of the pulmonary nodule revealed an adenocarcinoma.

## 3. Discussion

This case description highlights multiple life-threatening diagnoses emphasizing the complex interplay between the liver, pancreas, duodenum, and biliary system. The most notable clinical diagnoses described here are the duodenal perforation, multifactorial EHBO, and previously unreported hepatic neoplasia in a cat. Perhaps of equal clinical interest, the cat was primarily presented for severe signs most consistent with an extrahepatic biliary obstruction, and many of the comorbidities were unknown or unsuspected at the time of initial diagnostic investigation. It was not until the diagnostic process was well underway that it became evident that a multifactorial disease process was occurring and presented some of the most life-threatening and imminent concerns for the cat. Therein lies the unique paradigm and notable interests of this case for diagnosticians and clinicians in feline practice.

The cat in this report presented for evaluation with clinical signs (e.g. anorexia, lethargy, weight loss, and dehydration), laboratory abnormalities (e.g., hypoalbuminemia, hyperbilirubinemia, hypercholesterolemia, increases in ALP, ALT and GGT, and leukocytosis), and imaging findings consistent with the clinical description of EHBO described in the literature [1, 3, 5, 31, 32, 44]. Clinical presentations of feline EHBO are often chronic and nonspecific making achieving a diagnosis challenging and often result in extensive diagnostic investigations due to a lack of specificity [10, 32]. Treatment for EHBO often requires surgical decompression, biopsy, and/or biliary diversion. Criteria for surgical intervention is not completely defined. However, in this case, there was diagnostic evidence of a persistent obstruction which would have qualified the cat for surgical intervention.

Etiologies for feline EHBO are primarily neoplastic or inflammatory in nature including primary biliary or pancreatic adenomas or carcinomas as well as inflammatory bowel disease, enteritis, pancreatitis, cholangiohepatitis, bactobilia, cholelithiasis, and cholecystitis [3–5]. EHBO in this case was unique from the majority of previously reported EHBO cases as it was potentially caused by multiple contributing factors. This emphasizes the importance of complete patient evaluations ensuring concurrent contributory comorbidities are neither neglected nor missed, and the appropriate EHBO diagnosis is determined in order to pursue appropriate treatments. The cat's condition was complex due to the multiple comorbidities. The prognosis was considered grave and lacked a justifiable reason to recommend any surgical procedure. However, this case underscores the importance of clinicians understanding that atypical contributors to EBHO pathophysiology could be encountered at the time of surgery.

There were multiple contributing factors that likely played a role in the development of the EHBO including peritoneal inflammation, biliary carcinoma, and duodenal lymphoma at the sphincter of Oddi, neutrophilic cholangitis, and cholelithiasis. Necropsy evaluation confirmed the mechanical obstructive nature of the duodenal lymphoma, biliary carcinoma, and cholelithiasis. It was also suspected that the septic effusate resulted in intracavitary inflammation (peritonitis) in the area of the extrahepatic biliary tract. This presumptively acutely worsened the chronic cholangitis from an additional inflammatory stimulus worsening luminal narrowing and obstructive pathophysiology. All of the aforementioned disease processes induce variable degrees of cholestasis and might predispose to infection and inflammation in the hepatobiliary system and contribute to obstructions [2–4, 33–36].

Intraluminal biliary duct pathology further complicated this case by the presence of multiple choleliths contributing to the EHBO obstruction. Bile supersaturation, mucin hypersecretion, aberrant pH of bile, altered bile composition, cholecystitis, cholangitis, biliary stasis, aberrant biliary motility, dietary factors, hemolysis, and bactobilia (especially *E. Coli* and *Clostridium perfringens*) are all proposed etiologies for cholelith formation in cats [3–5, 34, 37, 38]. It is unknown what the cause of or type of the choleliths were in this case (testing at available evaluating institutions was suspended due to COVID-19 pandemic restrictions). Furthermore, it is unknown whether the neoplasia was a contributing factor to the biliary calculi EHBO, as it has not been reported, to the authors' knowledge, as a contributing factor for lithogenesis in cats. Choleliths as well as chronic cholecystitis have been shown to be risk factors for biliary carcinomas in humans [36, 39].

The duodenal perforation and septic abdomen were identified at necropsy and presumed to be present at the time of initial evaluation. Given the severity of this disease process, it is likely the leading cause of the cat's acute decline and presentation [40]. Published literature on duodenal perforation is limited due to its rare nature and is mostly extrapolated from human literature [41]. Two of the commonly cited underlying pathologies in duodenal perforation cases in people include both neoplastic causes as well as cyclooxygenase inhibition including corticosteroids and NSAIDs [11–13, 30, 41–43]. Infiltrative neoplasia can alter the integrity of the gastrointestinal wall and increase the risk of perforation of the bowel [9]. Corticosteroids and nonsteroidal anti-inflammatories alter the protective layer in the gastrointestinal tract by inhibiting enterocyte mucous production and prostaglandin-stimulated mucosal blood flow [14]. The limited reports of duodenal perforation in cats are often attributed to these same two etiologies in the majority of cases [15–24, 35]. Therefore, it is suspected that both the histopathologically diagnosed duodenal lymphoma and chronic steroid administration were contributing factors to the perforation in this case.

Another novel aspect of this case is the fact that this is the first reported unique double-negative phenotype of T-cell lymphoma (CD3+ and CD5+, but CD4- and CD8-) present in the duodenum and liver and suspected in the spleen. Hepatosplenic lymphoma has been previously reported in dogs [25] and a horse [26], but to the authors' knowledge, has not been described as a specific entity in the cat. Canine hepatosplenic lymphoma appears to frequently display a double negative (CD4-/CD8-) phenotype and is thought to have a worse clinical outcome than canine multicentric lymphoma [25, 27]. Feline gastrointestinal large granular lymphocyte (LGL) lymphoma has also been shown to occasionally display a double-negative phenotype; though, a CD8+ cytotoxic T-cell phenotype is more common [28]. However, no granules were observed in the present case; so, an LGL subtype is unlikely. A double-negative phenotype was also rarely observed in a study of cats with lymphocytosis, and among these cases, patients commonly had concurrent liver, spleen, gastrointestinal, and abdominal and peripheral lymph node involvement [45]. Thus, it is unclear whether hepatosplenic lymphoma is a separate entity in the cat, as in the dog and human, or an extension of disease in the gastrointestinal tract or bone marrow/peripheral blood. In the present case, there was no lymphocytosis (although flow cytometry was not performed on peripheral blood), and clinical and histopathologic features of lymphoma were much more prominent in the liver than in the GI tract, suggesting possible hepatic or hepatosplenic origin. Future evaluation of similar cases with complete clinicopathologic data may determine whether feline hepatosplenic lymphoma displays unique morphological features, immunophenotype, and clinical outcome.

## 4. Conclusion

Each of the seemingly disparate diagnoses described in the above description converge in this case leading primarily to two severe outcomes: EHBO and duodenal perforation. Independently, each of these conditions may cause severe biologic sequelae; in combination, they can be deadly. This discussion emphasized the importance of scrupulous diagnostic investigation and keen clinical awareness to ensure a holistic clinical approach to a seemingly straightforward case. This case embodies the importance of the fundamental medical principle referred to as Hickam's dictum.

## Data Availability

The data used in this case study can be provided on demand from the corresponding author upon request.
